# Orchestrating an Optimized Next-Generation Sequencing-Based Cloud Workflow for Robust Viral Identification during Pandemics

**DOI:** 10.3390/biology10101023

**Published:** 2021-10-11

**Authors:** Hendrick Gao-Min Lim, Shih-Hsin Hsiao, Yuan-Chii Gladys Lee

**Affiliations:** 1 Graduate Institute of Biomedical Informatics, College of Medical Science and Technology, Taipei Medical University, Taipei 11031, Taiwan; hendrick.san@gmail.com; 2 Division of Pulmonary Medicine, Department of Internal Medicine, School of Medicine, College of Medicine, Taipei Medical University, Taipei 11031, Taiwan; hsiaomd@gmail.com; 3 Division of Pulmonary Medicine, Department of Internal Medicine, Taipei Medical University Hospital, Taipei 11031, Taiwan

**Keywords:** next-generation sequencing, cloud computing, cloud workflow, pandemics, COVID-19, SARS-CoV-2, swine flu, H1N1

## Abstract

**Simple Summary:**

The recent infectious disease, coronavirus disease 2019, has become the novel pandemic event in the last decade after swine flu, which happened in 2009. While dealing with the pandemic, the challenge of gaining accurate identification results from abundant samples in a timely manner has still persisted. Here, in this study, we show the implementation of an optimized cloud workflow for a robust, yet accurate, identification process from these two latest pandemics events. This is a great example of how we integrate two current available technologies, next-generation sequencing and cloud computing, in practice into an applicable workflow for pandemics to tackle the issue of obtaining satisfactory results in a shorter time, while the abundant samples are available. Hopefully, the methods used in this study will intrigue more healthcare professionals to implement the cloud workflow as a part of the current identification method during the current or future pandemic and other infectious diseases as well.

**Abstract:**

Coronavirus disease 2019 (COVID-19), caused by severe acute respiratory syndrome coronavirus 2 (SARS-CoV-2), has recently become a novel pandemic event following the swine flu that occurred in 2009, which was caused by the influenza A virus (H1N1 subtype). The accurate identification of the huge number of samples during a pandemic still remains a challenge. In this study, we integrate two technologies, next-generation sequencing and cloud computing, into an optimized workflow version that uses a specific identification algorithm on the designated cloud platform. We use 182 samples (92 for COVID-19 and 90 for swine flu) with short-read sequencing data from two open-access datasets to represent each pandemic and evaluate our workflow performance based on an index specifically created for SARS-CoV-2 or H1N1. Results show that our workflow could differentiate cases between the two pandemics with a higher accuracy depending on the index used, especially when the index that exclusively represented each dataset was used. Our workflow substantially outperforms the original complete identification workflow available on the same platform in terms of time and cost by preserving essential tools internally. Our workflow can serve as a powerful tool for the robust identification of cases and, thus, aid in controlling the current and future pandemics.

## 1. Introduction

Novel coronavirus disease 2019 (COVID-19) has become a global pandemic since late 2019. The pandemic status was designated by the World Health Organization (WHO) in March 2020 because of the rapid increase in COVID-19 infection cases and deaths worldwide. COVID-19 has affected approximately 200 countries; more than 80 million people have been infected, and more than 1.8 million people have died within 1 year after COVID-19 was first reported in Wuhan, China, in late December 2019 [1,2]. In addition, COVID-19 is the second pandemic event to occur in the past decade after the swine flu pandemic, which occurred in 2009–2010 and affected countries worldwide, with estimated deaths ranging from 151,700 to 575,400 [3]. Therefore, developing an appropriate case identification strategy has become crucial while responding to pandemic events.

When talking about pandemics, the challenge of accurately identifying cases persists. The gold standard method used to identify cases of COVID-19 is the real-time reverse transcription-polymerase chain reaction (RT-PCR) for detecting the viral source [4], severe acute respiratory syndrome coronavirus 2 (SARS-CoV-2) that belongs to the following taxonomic order: family *Coronaviridae*, subfamily *Orthocoronavirinae*, genus *Betacoronavirus*, subgenus *Sarbecovirus*, and species *severe acute respiratory syndrome-related coronavirus* [5]. The same method was also applied to identify the last swine flu pandemic by detecting its source, the influenza A virus subtype H1N1, that belongs to a different *Orthomyxoviridae* family of viruses but still within the same *Orthornavirae* kingdom of viruses with SARS-CoV-2 [6]. Although RT-PCR is a well-established method for the detection of viral sources, it has a major shortcoming of a false-negative result because it relies on specific probe sequences [7,8]. As there are threats of accumulating mutations in the viral genome due to cross-species transmission [9,10,11], it is crucial whether modifications to the probe sequences are important to improve the confidence level of the PCR assay results. In this regard, an alternative metagenomics approach that utilizes next-generation sequencing (NGS) could be very useful because of the wide coverage of the viral sequences. Dissimilar to RT-PCR, which just observes probe target regions of the genome, NGS technology enables the ability to cover the entire length of the genome due to its power to sequence billions of nucleic acid bases simultaneously which is useful to confirm the identity of a specific virus, including one that presents in low numbers [12,13,14,15]. To achieve this purpose, the alignment of metagenomics sequencing data can be applied. When the reference genome was available, the typical alignment-based approach was performed by mapping sequencing reads against the genome to assess the taxonomic classification for every single read [16]. A variety of methods have been developed to support this purpose, ranging from early *MegaBLAST* [17] that utilized a seed search algorithm which is computationally infeasible nowadays due to the large size of sequencing data, popular *Kraken* [18] that implements other *k-*mer matches to align but still requires a lot of memory, to efficient *Centrifuge* [19] that takes less memory for alignment since it neither used seed search nor *k-*mer.

Another challenge when identifying a case during pandemics is handling the immense amount of data from a huge number of tested cases for both tested positive and negative. The job on analyzing this immense amount of data is an overwhelming task to perform by a human, even while using the RT-PCR that generates a relatively smaller data size for every sample compared to NGS. Meanwhile, a local personal computer provides limited computational power on processing the abundant data generated during pandemics, whereas cloud technology is able to process abundant data for performing rapid and scalable analyses [20]. On the other hand, orchestrating an analysis by integrating a running workflow with cloud computing can optimize the system performance, guarantee the quality of service, and reduce the running cost [21]. This approach provides a solution to overcome the large amounts of data generated by NGS and abundant test samples in terms of a pandemic event by providing more-powerful computational power and efficiency on performing analyses. The cloud enables an on-the-go metered services analysis to access shared pools of configurable computing resources via a network. In general, cloud services can be divided into four types: data as a service (DaaS), software as a service (SaaS), platform as a service (PaaS), and infrastructure as a service (IaaS). The application of each cloud service in the biological area utilizes public biological databases for DaaS, tools or workflows for SaaS, analysis and programming environments for PaaS, and virtualized resources such as a virtual central processing unit (vCPU) for IaaS [22].

In this study, we present a robust identification workflow in the cloud environment based on NGS data. Our workflow only needs raw NGS data as input files and fewer input parameter settings for it to run. In addition, our workflow automatically downloads a specific species of a pathogen reference genome as its index and utilizes cloud computing resources for its identification process. We hope that our simple yet robust workflow can become an option for anyone to identify cases rapidly and accurately in handling abundant samples during a pandemic.

## 2. Materials and Methods

### 2.1. Cloud Platform

We utilized the Cancer Genomics Cloud (CGC) platform [23] from Seven Bridges Genomics (SBG), Boston, MA, US A as the cloud technology to implement our workflow. The CGC provides many publicly available tools and workflows on their platform for performing reproducible analyses. In addition, as a public cloud platform, the GCG provides both genomics SaaS and PaaS that interface with another commercial cloud provider, Amazon Web Services (AWS), and the Google Cloud Platform as its IaaS [24].

### 2.2. Cloud Workflow

Our workflow was built on the basis of the *centrifuge* algorithm for the rapid, accurate, and sensitive classification of metagenomics sequences. *Centrifuge* is an indexing algorithm based on Burrows–Wheeler transformation [25] and the Ferragina–Manzini index [26], and it was applied for the efficient storage of reference genome sequences and the taxonomic mapping of metagenomics sequences. Instead of the *centrifuge* original classification purposes, our workflow was designed for robust identification purposes to target only specific reference genomes. Our workflow was built using the *Centrifuge* version 1.0.3.

**Figure 1:**
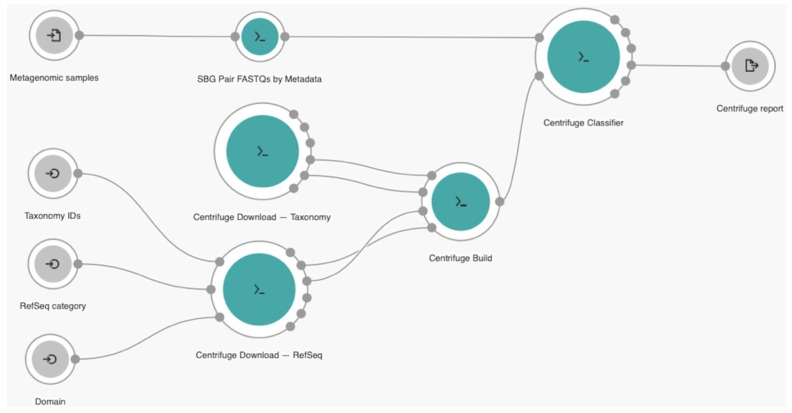
The proposed robust viral identification cloud workflow.

Our workflow was developed with *Rabix* [27] version 1.0.0 from SBG under Common Workflow Language (CWL) [28], that emerged as the workflow definition standard on creating a description of an analysis that is portable, scalable and support reproducibility across various software and hardware environments, ranging from workstations to clusters, cloud, and high-performance computing. *Rabix* is an integrated software environment to code, test, and debug CWL application. CWL application can be described as tool or workflow. A tool is a CWL description of an individual command line utility and its inputs and outputs. The tool can be executed independently or built into workflow, which is a collection of one or more connected tools. A workflow can also be the collection of other workflows. In *Rabix*, CWL application becomes nodes and edges to indicate the flow of data elements or variables between connected tools. The nodes work as individual or set of commands that can be executed in parallel, which can be the input, tool, or output. Meanwhile, edges represent data elements or variables, files or parameters, that pass from upstream to downstream nodes. The CWL workflow code can be described as the steps that can be exported as the JavaScript Object Notation (JSON) scheme. The visualization of our workflow that implemented on CGC platform using *Rabix* is shown in Figure 1. The icons 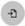, 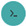, and 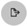 represent the input, tool, and output nodes, respectively.

In general, our workflow consisted of 4 main tools, 1 tool (*SBG Pair FASTQs by Metadata*) for quality control of input files and the rest 3 were the key features of *centrifuge* algorithm (*Centrifuge Download*, *Centrifuge Build*, and *Centrifuge Classifier*). At the beginning, the workflow takes **metagenomic samples** with the FASTQ file format which contains many metagenomic sequence reads and their quality scores as input. Then, *SBG Pair FASTQs by Metadata* tools preprocess these input files before the main identification process. At the same time, the *Centrifuge Download* tool fetches all necessary files required to build the index on the *Centrifuge Build* tool by parameterizing **taxonomy identifiers (IDs)** to list taxonomy identifiers of sequences that the user wants to download, **reference sequence (RefSeq) category** to filter database from which the sequences are downloaded and **domain** to download the specify domain that sequences can be downloaded with. Here, we used two different databases from the National Center for Biotechnology Information (NCBI): Taxonomy [29] and RefSeq [30]. Finally, the *Centrifuge Classifier* tool identifies the reads based on the index created previously. The final output of our workflow was *centrifuge* report for each metagenomics sample in TSV format, a tab-delimited text file with analysis results of each taxonomic category, such as *name*, *taxID*, *taxRank*, *genomeSize*, *numReads*, *numUniqueReads*, and *abundance*.

### 2.3. Experimental Setup

#### 2.3.1. Datasets

**Figure 2:**
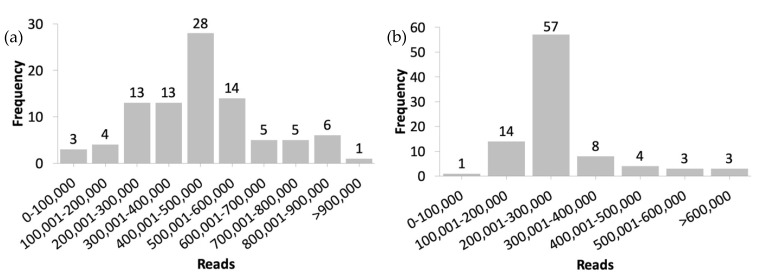
Read distributions of the (**a**) COVID-19 and (**b**) swine flu datasets.

We simulated the identification workflow process of the two most recent pandemic events that occurred in the world, namely, the current COVID-19 pandemic and the swine flu pandemic that occurred in 2009, by using open-access datasets. Two datasets listed in the NCBI BioProject [31] public repository were used to represent the two pandemic events: the accession code for COVID-19 was PRJNA625551, and the accession code for swine flu was PRJNA554447. In total, 65,944,624 reads from 182 samples with a file size of approximately 30 gigabytes (GB) that were registered before May 1, 2020, were used in this study, with 92 samples for COVID-19 and 90 samples for swine flu. All sequencing data were paired-end short reads generated using an Illumina MiSeq instrument with a viral ribonucleic acid (RNA) amplicon source. All original raw sequencing FASTQ files stored in the NCBI Sequence Read Archive (SRA) [32] public biological database were used as our input files. Table 1 summarizes the sequencing data profiles used in this study. The details of each sample used are available in the Supplementary File with the *Materials* tab prefix. The read distributions of both datasets are presented in Figure 2a for COVID-19 and Figure 2b for swine flu.

We also used the publicly available tool on the CGC platform called *SRA FASTQ-dump* fetch tool that adopted *SRA Toolkit* version 2.10.8. By running this tool in the cloud, all unsplit paired-end FASTQ raw files stored in the SRA database were directly transferred to the CGC platform by providing each file’s SRA accession code as the input.

#### 2.3.2. Parameterization

We used two settings when creating the *centrifuge* index to describe the viral source of each pandemic; that is, SARS-CoV-2 for COVID-19 and H1N1 for swine flu. Both settings used the same *reference genome* and *viral* type as input parameters for the **RefSeq category** and **domain**, respectively. However, we used different parameters to describe each species in **taxonomic IDs**: *2*,*697*,*049* for SARS-CoV-2 and *641*,*809* for H1N1 (A/California/07/2009). With the use of these settings, our workflow could retrieve original reference genomic sequences as FASTA files available in the RefSeq database for viral SARS-CoV-2 and H1N1 with genome sizes of 29,903 and 13,158 base pairs, respectively.

Finally, we used two settings of the AWS spot option that utilized the Amazon Elastic Compute Cloud: a c4.8×large compute-optimized instance that had a configuration of 36 vCPUs and 60 GB memory with 1024 GB of attached storage for running the *SRA FASTQ-dump* fetch tool as well as an r4.4×large memory-optimized instance that had 16 vCPUs and 122 GB of memory with the same size of attached storage for running the workflow.

#### 2.3.3. Evaluation Criteria

Several identification metrics were applied to assess the overall workflow performance. In the beginning, we defined cases and controls for each dataset depending on the index used. Subsequently, we used identified reads resulting from the *centrifuge* report and our unidentified read definitions to determine true positives (TPs), false positives (FPs), true negatives (TNs), and false negatives (FNs). Finally, we calculated the accuracy, sensitivity, and specificity of the overall workflow performance. Details of the definitions of the metrics used in this study are provided as follows:

Cases: All samples in the dataset that were highly correlated with the index used.

 

Controls: All samples in the dataset that were less correlated with the index used.

 

Total reads = “Number of spots” metadata information available in the SRA database for each sample.

 

Identified reads = Information regarding *numReads* from the *centrifuge* report available for each sample.

 

Unidentified reads = Total reads subtracted by identified reads.

 

TPs = Total number of identified reads in cases.

 

FPs = Total number of identified reads in controls.

 

TNs = Total number of unidentified reads in controls.

 

FNs = Total number of unidentified reads in cases.

 

(1) $$\mathrm{Accuracy}\;(\%)=\left[\frac{\mathrm{TPs}+\mathrm{TNs}}{\mathrm{TPs}+\mathrm{FPs}+\mathrm{TNs}+\mathrm{FNs}}\right]100.$$

(2) $$\mathrm{Sensitivity}\;(\%)=\left[\frac{\mathrm{TPs}}{\mathrm{TPs}+\mathrm{FNs}}\right]100.$$

(3) $$\mathrm{Specificity}\;(\%)=\left[\frac{\mathrm{TNs}}{\mathrm{TNs}+\mathrm{FPs}}\right]100.$$

Moreover, the following identification rate (IR) of a specific index was used as a single metric to evaluate the detailed workflow performance for each sample. In general, the IR was calculated by considering the definitions of our previously identified reads and total reads. However, when calculating the individual TP, FP, TN, and FN of each sample for both cases and controls, the IR was defined as follows:

(4) $$\begin{array}{cc} \mathrm{IR}\;(\%) & =\left[\frac{identified\;reads\;}{total\;reads}\right]100\\ & =\left[\frac{TP\;}{TP+FN}\right]100\;(\mathrm{true}\;\mathrm{positive}\;\mathrm{rate},\;\mathrm{for}\;\mathrm{each}\;\mathrm{case})\;OR\\ & =[\frac{FP\;}{TN+FP}]100\;(\mathrm{false}\;\mathrm{positive}\;\mathrm{rate},\;\mathrm{for}\;\mathrm{each}\;\mathrm{control}). \end{array}$$

## 3. Results

Table 2 summarizes all metrics used in this study. Details of TPs, FPs, TNs, and FNs along with the IR for each sample used in this study using both SARS-CoV-2 and H1N1 indices can be found in the Supplementary File with the *Results* tab prefix.

**Figure 3:**
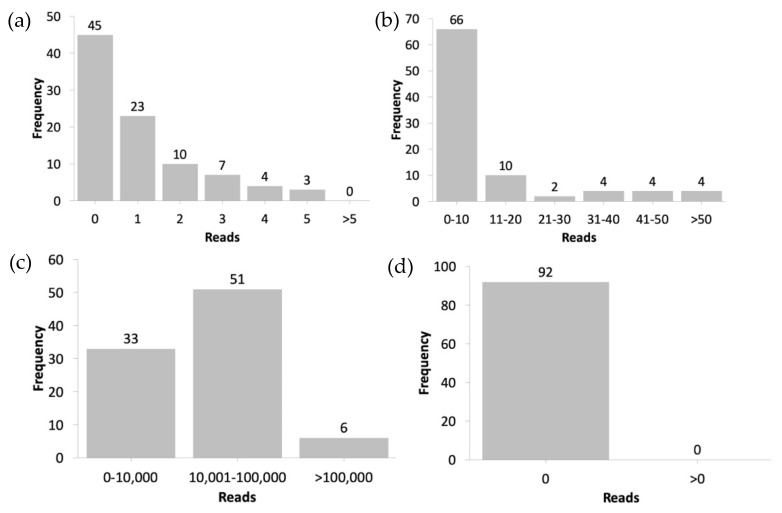
Number of (**a**) unidentified read(s) for each sample in the COVID-19 dataset and (**b**) identified read(s) for each sample in the swine flu dataset when using SARS-CoV-2 as the index within number of (**c**) unidentified reads for each sample in the swine flu dataset and (**d**) identified read for each sample in the COVID-19 dataset when using H1N1 as the index.

When SARS-CoV-2 was used as the index for all samples, our workflow demonstrated a satisfactory accuracy, sensitivity, and specificity, with an IR of 99.999–100% for the COVID-19 dataset and 0–0.033% for the swine flu dataset. The majority of samples in our COVID-19 dataset (45) had an IR of 100% with zero unidentified reads (Figure 3a), whereas 30% of total samples in our swine flu dataset (27) had zero identified reads, and the remaining samples exhibited varying numbers of identified read(s), from 1 to 180 (Figure 3b). However, we did not observe the same results when using H1N1 as the index. The sensitivity of our workflow decreased to 10% because various ranges of IRs were detected in swine flu dataset samples (37–98%), resulting in various unidentified reads (Figure 3c), even though the workflow demonstrated a specificity of 100% when all 92 samples in the COVID-19 dataset had an IR of 0% with zero identified reads (Figure 3d). This disparity may be attributed to the difference in the PCR primer design used to target specific viral rather than off-target viral sequences [33]. On the basis of dataset metadata information available in the SRA database, the influenza A universal type primer was used to target viral RNA in the swine flu dataset. The use of this primer enabled the detection of other subtypes of influenza A virus apart from H1N1. Influenza A has many subtypes based on its antigenic characterization of two proteins on the virus surface: hemagglutinin (H) and neuraminidase (N) [34]. Until now, 18 hemagglutinin subtypes, H1–H18, and 11 neuraminidase subtypes, N1–N11, have been identified. Moreover, in the RefSeq database, apart from the H1N1 (A/California/07/2009) subtype we used in this study, the reference genomes of the following three subtypes of influenza A virus were available: H2N2 (A/Korea/426/1968), H3N2 (A/New York/392/2004), and H7N9 (A/Shanghai/02/2013). On the other hand, we could also set the threshold on the IR to determine the infection of a certain viral species type. Taking our experiment of using SARS-CoV-2 species as an index as an example, if the IR was considerably high (>99%) in the sample (COVID-19 dataset), then that sample could be considered to be infected by this species. Moreover, if the IR was considerably low (<0.05%) in the sample (swine flu dataset), then that sample could be considered to not be infected by this species.

## 4. Discussion

### 4.1. Principal Results

We developed our optimized NGS-based cloud workflow by using three major tools of the *centrifuge* algorithm: *Centrifuge Download*, *Centrifuge Build*, and *Centrifuge Classifier*. Our workflow was a compact and straightforward representation of complete metagenomics *centrifuge* workflows that can be used for case identification on the CGC platform. For the rapid identification of cases, we focused only on generating the main *centrifuge* report as our output to determine the number of input sample reads mapped to specific species. In our experiment using open-access datasets, our workflow had satisfactory overall and detailed performance results depending on the reference genome used as its index.

Our workflow can be advantageous for researchers who have limited computational resources and even general users with a limited programming background because it provides a user-friendly interface for performing the identification process. By contrast, running the same *centrifuge* algorithm as a standalone program on a local computer requires prior programming knowledge. Our workflow interface makes the identification process easier by providing only sequencing files. Moreover, few parameter settings are required to run our workflow without the need to run line-by-line of the algorithm code. In addition, by running the analysis in the cloud enables flexibility in configuring resources, which cannot be performed on a local computer with a fixed system configuration, thereby facilitating the scaling of analyses depending on the requirement. Taking our experiment as an example, we utilized two types of spot instances on the CGC platform: compute-optimized instances for running a fetch tool and a memory-optimized instance for running the workflow. In addition, resources can be switched to another instance that has more computational power to support the identification of numerous samples during a pandemic. Furthermore, if people want to use their own sequencing datasets stored in the local computer, the data should be in a FASTQ file format and can be transferred into the CGC platform using the desktop uploader tool provided by SBG called *CGC Uploader,* which works across many operating system such as Windows, Mac, or Linux.

### 4.2. Limitations

Choosing an appropriate algorithm was also another key factor affecting the efficiency of the workflow. In addition to *centrifuge*, many algorithms are available for classification purposes such as *MegaBLAST*, *Kraken*, *CLARK* [35], *metaOthello* [36], *taxMaps* [37], and *PathSeq* [38]. However, Ye et al. [39] reported that *centrifuge* is 1) powerful when used with a database that is not loosely compressed, such as the RefSeq database, because *centrifuge* discards original database sequences to save space and 2) requires relatively less time and memory to run while maintaining good classification results. We demonstrated *centrifuge* as one of the metagenomics algorithms that uses the RefSeq database to create its own custom-built reference genome index for the robust identification purpose. In addition, as long as other algorithms have their own raw source code available online, they can also be possibly implemented in the CGC or even other cloud platforms. Furthermore, unlike the *centrifuge* original publication that targeted multiple species for classification purposes, our *centrifuge-*based cloud workflow with settings that were optimized for the identification process guarantees robustness by taking one specific species into account. By doing this, the *centrifuge* can be used to analyze a huge number of samples in a shorter time since it just considers one specific reference genome of one species which will not take much time to do so compared to the original one that uses multiple reference genomes of multiple species.

Our workflow that was built using CWL also followed Findable, Accessible, Interoperable, and Reusable (FAIR) principles [40] to ensure reproducibility, reusability, and transparency for its use by the public. Our workflow script code under the JSON format applied in this paper is publicly accessible (Findable and Accessible) and can be implemented on the CGC platform (Interoperable and Reusable). Our code can also be executed using other CWL executors, such as *CWL-Tool* and *CWL-Airflow* [41]. However, because the code contained a platform-specific field, it might not work outside the CGC platform. To use our CWL code, an account on the CGC platform would be required in advance.

### 4.3. Species Identification at a Lower Taxonomy Level

To test the sensitivity of our workflow for identifying species with higher similarities with SARS-CoV-2 at the lower taxonomy level, we conducted another experiment by using Middle East Respiratory Syndrome Coronavirus (MERS-CoV) sequencing data. We chose MERS-CoV because it has a close similarity to the genus *Betacoronavirus* of SARS-CoV-2, but has a different lineage. SARS-CoV-2 belongs to lineage B (subgenus *Sarbecovirus*), whereas MERS-CoV belongs to lineage C (subgenus *Merbecovirus*) [42,43]. In addition, the outbreak of MERS-CoV occurred worldwide in 2012 [44].

Data used to represent MERS-CoV were also obtained from the same public BioProject repository, with an accession code of PRJNA316178. We tested a total of eight samples by using our workflow and the same parameterization settings; however, we used only SARS-CoV-2 as the index. In addition, we replaced the H1N1 index with the MERS-CoV index. The following input parameter settings were applied to describe MERS-CoV: *reference genome* for **RefSeq category**, *viral* for **domain**, and *1*,*335*,*626* for **taxonomy IDs**. Under these settings, our workflow could generate the reference genome sequences of the Middle East Respiratory Syndrome-related coronavirus (MERS-related type) as FASTA files with a length of 30,119 base pairs. Table 3 summarizes MERS-CoV dataset profiles, and the details of the dataset within each identification result are provided in the Supplementary File with the tab name *Discussion_MERS-CoV*.

The MERS-CoV dataset exhibited characteristics similar to those of our previous two pandemic sequencing datasets, in which paired-end short reads were generated using the Illumina MiSeq instrument with a viral RNA source. However, the average number of reads available for this MERS-CoV was higher than those in our other two pandemic datasets. This difference can be attributed to the difference in the sequencing strategy used, wherein dataset metadata were referred. In contrast to our pandemic datasets which used the amplicon strategy, whole-genome shotgun (WGS) was used for our MERS-CoV dataset. WGS generated a higher number of reads than the amplicon strategy did because WGS covers a wider portion of the genome within a sample instead of only a partial part of the genome, such as an amplicon [45,46]. This finding also explains why the ratio of the average file size to the average number of reads in the MERS-CoV dataset (~6 × 10^−4^) was higher than those of our two pandemic datasets (approximately 4 × 10^−4^ for the COVID-19 dataset and 5 × 10^−4^ for the swine flu dataset). This ratio was useful to illustrate the amount of information stored in each read on average. However, since the ratio was close to that of both pandemic datasets, we could still conclude that these datasets (COVID-19, swine flu, and MERS-CoV) had similar characteristics for short-read sequencing data.

**Figure 4:**
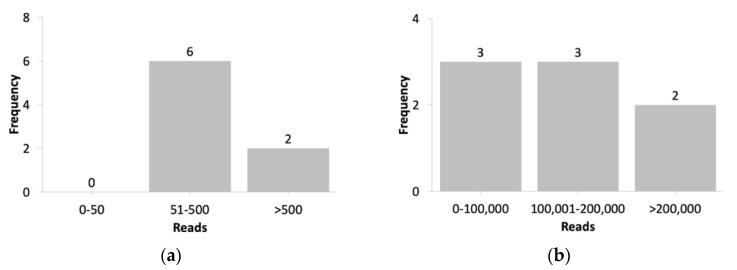
Number of reads (**a**) identified as SARS-CoV-2 when using the SARS-CoV-2 index and (**b**) unidentified when using the MERS-CoV index in the MERS-CoV dataset.

In this experiment, the MERS-CoV dataset generated a higher number of reads that were identified as SARS-CoV-2 (Figure 4a), resulting in an IR of 0.008–0.06%. By contrast, when using the same SARS-CoV-2 index, we obtained an IR of 0–0.033% in the swine flu dataset. These results suggest that sequencing data obtained from MERS-CoV, which shares a higher similarity with SARS-CoV-2, identified a higher number of reads for SARS-CoV-2 compared with sequencing data obtained for H1N1 that shared a lower similarity with SARS-CoV-2. When we used the MERS-related-type index, our workflow exhibited an IR of 77–99%, with its number of unidentified reads (Figure 4b) being higher than those obtained using the swine flu dataset with H1N1 as the index (IR: 37–98%). This result can be attributed to the use of a primer specific to MERS-CoV based on limited MERS-CoV metadata information available in the database. In addition, the low specificity of the index used in our workflow could be another factor that could have resulted in various IR range results in our MERS-CoV dataset.

### 4.4. Comparison with Prior Work

**Figure 5:**
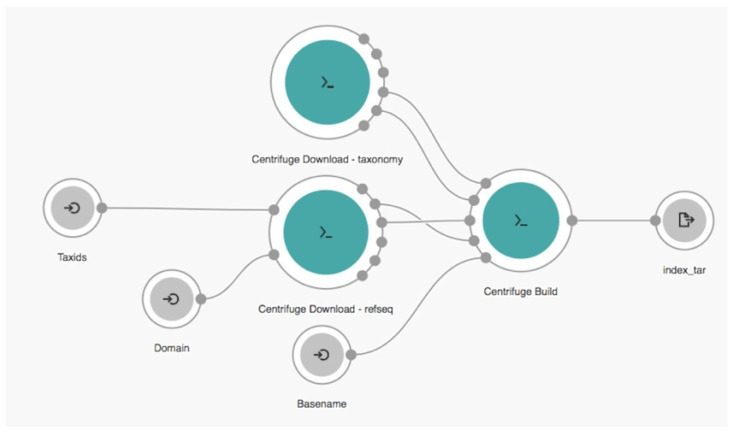
Original Reference Index Creation—Centrifuge 1.0.3 sub-workflow.

**Figure 6:**
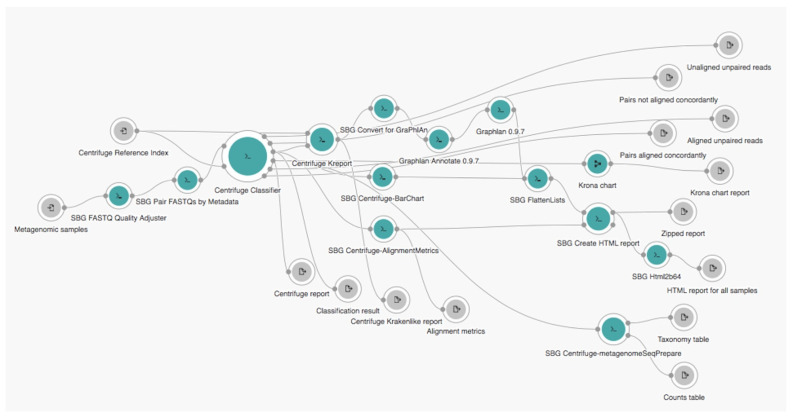
Original Metagenomics WGS analysis—Centrifuge 1.0.3 sub-workflow.

We compared the performance of our workflow with that of the original complete *centrifuge* workflow available on the CGC platform that contains two independent *centrifuge* sub-workflows, namely, the *Reference Index Creation—Centrifuge 1.0.3*—and *Metagenomics WGS analysis—Centrifuge 1.0.3*—as shown in Figure 5 and Figure 6, respectively. The same input files of pandemic datasets and same parameter settings were applied for both workflows.

Because our workflow used the same *centrifuge* algorithm such as the original complete *centrifuge* workflow available on the CGC platform, our workflow also generated the same *centrifuge* report as that generated by the original workflow. However, unlike the original workflow that generated many additional output files from many other tools inside (e.g., *Kreport*, *Graphlan*, and *Krona*), our workflow only retained a *centrifuge* report directly generated by the *centrifuge* algorithm to summarize the number of reads identified in a specific index; thus, the report was sufficient to perform a robust identification. In addition, within our workflow, users do not need to manually pass the result from one sub-workflow to another again such as the original one, since this process was performed automatically. This difference made our workflow work very optimally, which implicate to a reduced running time as well cost since the cost was linearly counted based on a time basis.

We used the following formula to calculate the computational efficiency (Eff) achieved by our workflow in terms of time (T) in minutes (min) and cost (C) in United States Dollar (USD) compared with the original complete *centrifuge* workflow under the same parameter settings:

(5) $$\mathrm{Eff}\;(\%)=\left[\frac{\;\Delta T\;or\;C\;between\;original\;and\;our\;workflow}{\;\;\sum T\;or\;\;C\;taken\;by\;original\;workflow}\right]100;$$

where ΔT or C indicate the difference in time or cost between the original *centrifuge* workflow and our workflow and ∑T or C indicates the total time or cost required to run the two sub-workflows of the original complete *centrifuge* workflow. The results are listed in Table 4.

The time and cost required for running the *Reference Index Creation* sub-workflow in the original workflow were 2 min and USD0.02, respectively, when using both SARS-CoV-2 and H1N1 indexes. However, the time and cost required for running the *Metagenomics WGS analysis* sub-workflow in the original workflow was 33 min and USD0.31, respectively, when using the SARS-CoV-2 index and 47 min and USD0.43, respectively, when using the H1N1 index. Our identification workflow demonstrated an efficiency of more than 70% in terms of both time and cost when using both indexes. This result indicated that the time and cost of our identification workflow were considerably lower than those of the original workflow. Here, we excluded the time and cost required to fetch all the raw input files of 182 samples by using the *SRA FASTQ-dump* fetch tool in one round (53 min for USD0.62) prior to running all workflows.

## 5. Conclusions

Integrating two currently available technologies, next-generation sequencing and cloud computing, can be powerful for the viral identification purpose in the current COVID-19 pandemic and even future pandemics. The application of our robust identification workflow to the cloud by using next-generation sequencing data as its input demonstrated the feasibility of merging these two technologies with good synergy for handling pandemic events. Our workflow was successfully implemented in the cloud and could distinguish two pandemics that occurred in the world over the last decade based on next-generation sequencing data. Our workflow can be a favorable approach for simple, scalable, accurate, affordable, robust, and reproducible case identification during a pandemic.

In the future, our cloud workflow capability can be extended, not only limited to identify the virus itself, but also to detect the type of mutation with respect to the emerging variants of the current pandemic situation. Furthermore, we hope that our studies can encourage many healthcare professionals to apply the cloud workflow into their workspace, especially for the automation of the viral identification purpose.

## Figures and Tables

**Table 1:** Summary of sequencing data profiles.

Dataset	Reads				File Size (in Megabyte)			
	Total	Minimum	Maximum	Average	Total	Minimum	Maximum	Average
COVID-19	42,210,829	27,707	1,208,398	458,813	16,621	7	587	181
Swine flu	23,733,795	95,755	633,535	263,709	12,715	49	357	141

COVID-19, coronavirus disease 2019.

**Table 2:** Summary of workflow identification results.

Metric	Index	
	SARS-CoV-2	H1N1
True positives	42,210,734	21,341,623
False positives	967	0
True negatives	23,732,828	42,210,829
False negatives	95	2,392,172
% Accuracy	99.9984	96.372
% Sensitivity	99.9998	89.921
% Specificity	99.9959	100.000
% IR range (cases)	99.999–100	37–98
% IR range (controls)	0–0.033	0

SARS-CoV-2, severe acute respiratory syndrome coronavirus 2; IR, identification rate.

**Table 3:** MERS-CoV sequencing data profiles.

	Total	Minimum	Maximum	Average
Reads	8,883,737	570,733	3,141,921	1,110,467
File size (in megabyte)	5713	370	2000	714

**Table 4:** Time and cost comparisons of running workflows.

Workflow (Index Used)	Time (min)	Time Efficiency	Cost (USD)	Cost Efficiency
Original workflow (SARS-CoV-2)	35 (2 + 33)	71%	0.33 (0.02 + 0.31)	76%
Our workflow (SARS-CoV-2)	10		0.08	
Original workflow (H1N1)	49 (2 + 47)	82%	0.45 (0.02 + 0.43)	84%
Our workflow (H1N1)	9		0.07	

min, minutes; USD, United States Dollar.

## Data Availability

The workflow source code is freely available for reproducible purposes on the Cancer Genomics Cloud platform at https://github.com/hendrick0403/NGSbasedWorkflow. Meanwhile, both pandemics (COVID-19 and swine flu) and other MERS-CoV datasets used in this study can be found in the NCBI BioProject public repository (https://www.ncbi.nlm.nih.gov/bioproject/) with accession code of PRJNA625551, PRJNA554447, and PRJNA316178, respectively.

## Supplementary Materials

The following are available online at https://www.mdpi.com/article/10.3390/biology10101023/s1. Detailed information of all datasets used in this paper and their identification performance are available in a Supplementary Excel File.
